# Restorative Management and Treatment of Pseudo-Class III Malocclusion

**DOI:** 10.1155/2021/8470222

**Published:** 2021-11-03

**Authors:** Ali H. Alfaifi

**Affiliations:** King Khalid University, College of Dentistry, Department of Prosthodontics, Abha, Saudi Arabia

## Abstract

**Introduction:**

One of the challenges that clinicians encounter in the dental office is treating occlusal discrepancies. Malocclusion has been classified into three main classes that were further classified by researchers into more detailed subclasses later on. Pseudo-class III malocclusion has been called apparent or positional class III malocclusion, and its treatment usually consists of different modalities depending on how early the case is treated. When early orthodontic intervention was not possible, the restorative treatment becomes an excellent alternative especially when the teeth require restorative rehabilitation. *Treatment*. In this case report, the patient was suffering from dental wear, multiple failed old restorations, and edge-to-edge occlusal relationship which could be classified as pseudo-class III malocclusion. The patient's esthetic complaint was addressed with full-coverage lithium disilicate and monolithic zirconia restorations that were successful in reestablishing the patient's occlusal relationship and were able to eliminate the biological manifestations of dental caries.

**Conclusion:**

We were able to provide an alternative to orthodontic treatment where esthetic and functional needs of the patient were met after careful diagnosis and proper management. This clinical approach will give chance to treat patients suffering from minor occlusal discrepancies that require restorative intervention without the need to go through orthodontic therapy.

## 1. Introduction

Angle classified malocclusion based solely on the dental components, which gave a chance for more detailed classifications to come later to accommodate the variabilities encountered by clinicians every day [1, 2]. The newer classifications were able to accommodate the differences between different manifestations of the same original class of malocclusion. Generally, class III malocclusion or mesioocclusion could be divided into skeletal and pseudo-class III [2]. Pseudo-class III malocclusion is different from the skeletal type in that the skeletal components of the maxilla and the mandible are in a normal class I relationship (orthognathic), but the difference is in the dental components in which the teeth might be in a class III relationship [2, 3].

The prevalence of class III malocclusion is very common among Asian populations, including Korean, Japanese, and Chinese, with an estimated incidence rate of 4-14%. The number is much less among Caucasians and African populations [3, 4]. Pseudo-class III malocclusion is also called positional class III malocclusion due to the anterior displacement of the mandible in relation to the maxilla which is characterized by an anterior crossbite with a reverse horizontal overlap (negative overjet), retroclined maxillary anterior teeth, and proclined mandibular anterior teeth [5, 6].

The etiology behind this functional discrepancy is usually attributed to multiple factors like early loss of deciduous teeth and ectopic eruption of permanent dentition. Functionally, it is believed to be because of abnormality in tongue position and/or airway problems. In addition, there might be minor skeletal discrepancies in the maxilla that could lead to this type of malocclusion [7]. It is not uncommon to encounter patients in the dental office with occlusal discrepancies that require both orthodontic and restorative treatment. Patients are usually discouraged to commit to the orthodontic therapy as it might extend for long periods of time and this will make them eventually explore the restorative option. It was our goal in this case report to illustrate the restorative option in treating minor cases of pseudo-class III malocclusion and to discuss the challenges and steps involved in the treatment process.

## 2. Case Report

### 2.1. Chief Complaint and Diagnosis

**Figure 1:**
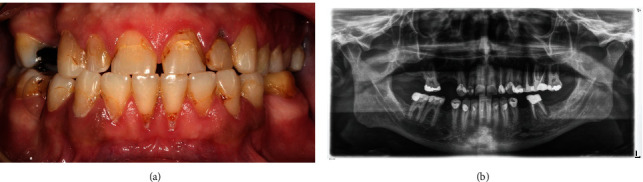
(a) Intraoral frontal view of the patient teeth preoperatively. (b) Panoramic X-ray view of the patient teeth preoperatively.

Thirty-one-year-old Middle Eastern male presented at the prosthodontic clinic in a private office in Abha, Saudi Arabia, with a chief complaint“ I need to have a better smile.” The patient had no contributory past medical history except smoking a pack of cigarettes daily. Extraoral examination revealed no abnormality including TMJ, lymph nodes, facial symmetry, and lateral profile. Intraorally, the patient had multiple old discolored composite restorations in the anterior area in both arches with recurrent caries underneath. The patient had wear of the maxillary anterior teeth and had multiple missing teeth with deficient alveolar ridges. He also had a large old restoration on #22 with recurrent caries that caused recession of the gingiva apical to the tooth. Additionally, large old restorations on multiple posterior teeth were noted with periapical radiolucency on tooth #36 related to an old root canal treatment. The patient also had midline diastema and retroclined maxillary anterior teeth with a normal molar relationship. The anterior tooth malposition was expressed by the patient as a concern when he smiles. Periodontal status of anterior teeth was fair with minimal bone loss. However, localized horizontal bone loss was evident in the posterior area, especially in the maxillary right posterior area with maxillary sinus pneumatization (Figure 1).

Diagnosis included pseudo-class III malocclusion accompanied by dental wear of maxillary anterior teeth and midline diastema. He also had recurrent caries related to the old defective composite restorations in both maxillary and mandibular anterior teeth. Additionally, the patient had recurrent caries on multiple maxillary and mandibular posterior teeth with periapical radiolucency related to tooth #36. After diagnosis and treatment plan presentation, the patient agreed on extracting tooth #22 as it was deemed unrestorable. As the patient wanted to improve his smile and correct his malocclusion with no orthodontic intervention, replacing his anterior teeth with full-coverage lithium disilicate restorations was planned. The missing teeth #14 and #15 also were going to be replaced with a zirconia fixed dental prosthesis as the patient did not want to go through implant treatment. The patient was not interested in treating the mandibular teeth and wanted to treat his maxillary anterior teeth only as an initial stage.

### 2.2. Treatment Progress

**Figure 2:**
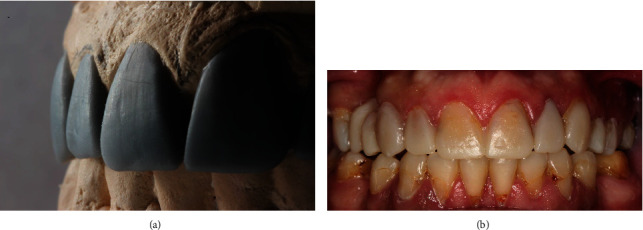
(a) Diagnostic wax-up illustrating the desired corrected tooth relationship. (b) Diagnostic mock-up intraorally.

Maxillary and mandibular irreversible hydrocolloid impressions were made, and proper interocclusal records were taken. All impressions and records were sent to the lab for diagnostic wax-up and for silicone matrix fabrication for diagnostic mock-up. At the mock visit, esthetics, phonetics, and function were initially evaluated and were confirmed satisfactory (Figure 2). The patient was then referred to an oral surgeon to have his tooth #22 extracted after initial preparation of anterior maxillary teeth to facilitate the fabrication of the provisional FDP to replace tooth #22 immediately after extraction. An Ovate Pontic was made to facilitate tissue sculpturing of the extraction site on #22 to maintain esthetic outcomes.

**Figure 3:**
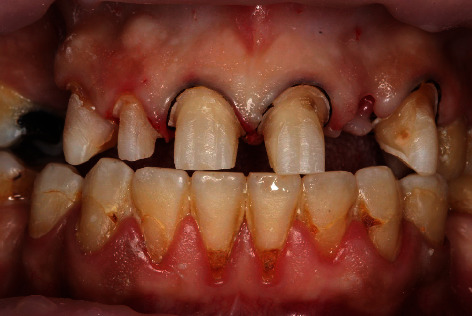
The extraction site with the maxillary anterior teeth after preparation at the follow-up appointment two weeks after extraction.

**Figure 4:**
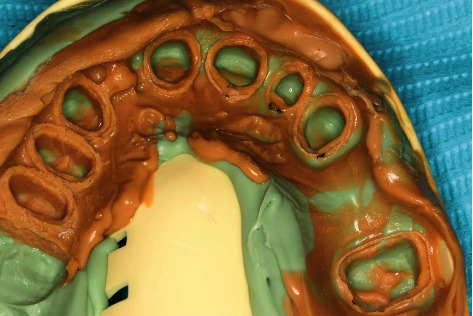
Final impression after tooth preparation using vinyl polysiloxane impression material.

**Figure 5:**
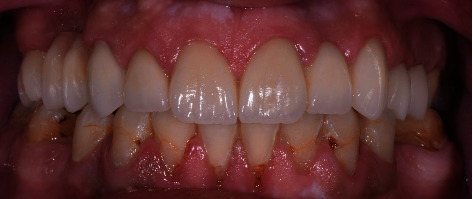
Postoperative intraoral frontal view of the full-coverage restorations illustrating the corrected occlusal relationship.

The patient was seen two weeks postextraction for a follow-up to evaluate the tissue maturation. After removing the maxillary anterior provisionals, the tissue maturation around the extraction site was confirmed satisfactory (Figure 3). The remaining maxillary teeth from #16-25 were prepared. Then, gingival displacement was performed using the double retraction cord technique proceeded by making the final impression (Figure 4) using light- and medium-body vinyl polysiloxane impression material (3M Imprint; United States). The final restorations were made to adopt the patient's existing occlusal scheme as the anterior edge-to-edge relationship did not require changing the occlusal vertical dimension as confirmed by the provisional restorations. Missing teeth #14 and#15 were replaced by monolithic zirconia FDP, while teeth from #12 to #25 were designed to have lithium disilicate full-coverage restorations, including the FDP replacing #22 (Figure 5).

## 3. Discussion and Conclusion

It is very common for a patient with malocclusion problems to seek the restorative alternative as orthodontic treatment would take extended periods of time. In this paper, our goal was to show that in minor cases of apparent class III malocclusion, we can explore solving the patient esthetic concerns by utilizing full-coverage restorations, especially when the teeth' existing conditions necessitate the restorative alternative and when the periodontal condition of the involved teeth is acceptable [8–10].

It is critical to accurately diagnose malocclusion conditions and differentiate between what could be a skeletal abnormality from any other functional discrepancies. In minor cases of pseudo-class III malocclusion, it is important to examine the patient's dentition and take proper occlusal and centric records to confirm the right diagnosis and be able to execute the appropriate treatment plan. In this paper, it was not necessary to change the OVD as the occlusal discrepancy was minor, and the patient went through a provisional phase where no complications were observed regarding the new occlusal relationship of the maxillary and mandibular teeth, especially in the anterior region. The periodontal health of the teeth involved in malocclusion treatment was confirmed satisfactory. Proper examination in these cases is very important as clinicians might feel pressured by their patients to conform to the restorative treatment of malocclusion when it is possible to treat the patient with orthodontics only preserving tooth structure and avoiding over treating patients without proper justification. In this case, the restorative needs and the fair periodontal condition of the patient's anterior teeth were important prerequisites before proceeding with the patient decision to correct his smile restoratively.

**Figure 6:**
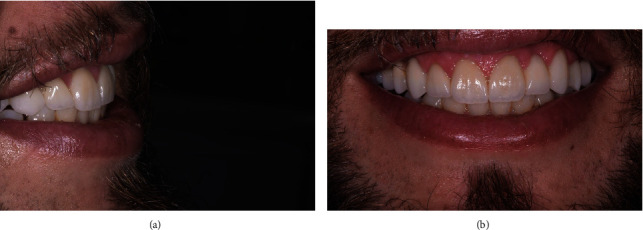
(a) Extraoral lateral view of the final restorations illustrating the desired esthetic outcome. (b) Extraoral frontal view of the patient smile with the final full-coverage restorations.

This paper illustrated a restorative alternative in correcting minor occlusal discrepancies classified as pseudo-class III malocclusion. With the patient's specific needs and careful clinical evaluation, it was decided to correct the patient's malocclusion with full-coverage restorations. The patient's chief complaint was addressed from an esthetic point of view where his edge-to-edge relationship, midline diastema, and old defective restorations were corrected utilizing full-coverage lithium disilicate and monolithic zirconia restorations that yielded satisfying esthetic outcomes (Figure 6). Functionally, the patient's new occlusal relationship, especially in the anterior area, needs to be evaluated periodically as it is important to observe any complications that might happen postoperatively and address them accordingly. This clinical approach will give chance to treat patients suffering from minor occlusal discrepancies that require restorative intervention without the need to go through orthodontic therapy.

## Data Availability

No data were used to support this article.
